# Male bumblebees sustain mate-seeking by adjusting foraging to environmental conditions

**DOI:** 10.1093/beheco/arag054

**Published:** 2026-05-19

**Authors:** Natacha Rossi, Charlotte Doussot, Joseph L Woodgate, Mathieu Lihoreau, Lars Chittka

**Affiliations:** Ecology and Evolution, School of Life Sciences, University of Sussex, Falmer, Brighton BN1 9RH, United Kingdom; Research Centre on Animal Cognition (CRCA), Centre for Integrative Biology (CBI); CNRS, Toulouse University, 31062 Toulouse Cedex 9, France; School of Computer Science, University of Sheffield, Sheffield S1 4DP, United Kingdom; Research Centre on Animal Cognition (CRCA), Centre for Integrative Biology (CBI); CNRS, Toulouse University, 31062 Toulouse Cedex 9, France; Centre for Brain and Behaviour, School of Biological and Behavioural Sciences, Queen Mary University of London, London E1 4NS, United Kingdom

**Keywords:** behavioral plasticity, *Bombus terrestris*, movement ecology, pollinator, reproductive signaling

## Abstract

Male bees navigate complex tradeoffs between energy acquisition and reproductive signaling, yet their movement strategies remain understudied. Unlike workers that optimize foraging to support the colony, male bumblebees (*Bombus terrestris*) forage independently to collect nectar and deposit sex pheromones on selected plants. Using high-resolution 3D tracking in an indoor flight cage, we investigated how the spatial arrangement of nectar and scent-marking sites, along with nectar availability, influence male movement patterns. We manipulated the distribution of feeders (artificial flowers) and scent-marking locations (branches), and varied nectar delivery rates, to assess effects on foraging, scent-marking, and patrolling. Males responded strongly to spatial structure: in clumped arrays with evenly spaced resources, movements between consecutive visits were shorter and more localized, while in dispersed arrays with irregular spacing, transitions were longer and more variable. The combination of dispersed spacing and low nectar availability imposed the highest foraging demands, resulting in fewer feeding events and reduced total feeding time. Despite these increased costs, males maintained consistent investment in reproductive behaviors, suggesting a prioritization of mate-seeking over energy gain. Rather than reducing signaling, males adjusted their foraging strategy—favoring fewer but prolonged feeding bouts when nectar availability allowed. These findings reveal a unidirectional behavioral adjustment, in which foraging is modulated to sustain reproductive effort, and show how spatial resource structure and nectar availability together shape movement decisions in male pollinators.

## Teaser

Male bumblebees keep searching for mates even when food is scarce, adjusting how they forage to conserve energy. Using 3D tracking in a controlled environment, we found that males reduce foraging effort but maintain reproductive behaviors. This shows how animals balance survival and reproduction in changing environments, offering new insights into pollinator behavior and resilience.

## Introduction

Animals adjust their allocation of time and energy in response to internal state and external pressures, shifting between foraging and reproduction as conditions change (Gavassa et al. 2013; Dougherty 2021; Westwood et al. 2025). For males in particular, behavioral decisions, such as when and where to forage or engage in reproductive signaling, often reflect tradeoffs between energy acquisition and mating effort. When resources are scarce, males may prioritize energy acquisition (Billings et al. 2019). However, when resources are abundant or competition for mates is high, they may shift their investment towards mate-seeking (Meuche and Grafe 2009; Höbel 2015; Fernlund Isaksson et al. 2022; Wilde et al. 2023).

Movement strategies thus often reflect the functional demands of a given behavior, whether it be foraging, defending resources, or seeking mates. For instance, hummingbirds defend fixed feeding territories to ensure exclusive access to high-reward resources that can be monopolized (Sargent et al. 2021). In contrast, butterflies move more randomly when searching for mates, a strategy suited to locating widely dispersed and unpredictable mates in open environments (Reynolds 2006). Many nectar-feeding animals, including bees, establish stable, repeatable routes between feeding locations (traplines), which maximize energy efficiency and minimize travel costs in environments where resources are spatially stable but not defensible (Mailly et al. 2025).

Bumblebee workers (*Bombus* spp.) exemplify route optimization: they learn sequences of flower visits that they adjust in response to changes in spatial layout and nectar availability (Cartar and Real 1997; Chittka et al. 1997; Thomson et al. 1997; Ohashi et al. 2007; Raine and Chittka 2007; Ohashi and Thomson 2009; Lihoreau et al. 2012; Woodgate et al. 2017). However, male bumblebees follow a fundamentally different life history trajectory. Soon after eclosion from their pupae, males typically leave the nest permanently, no longer contributing to colony tasks such as foraging or brood care (Free 1982; Kearns and Thomson 2001). Instead, they roam independently, balancing the need to forage for themselves with the imperative to locate and attract mates (Frank 1941; Haas 1946; Haas 1949; Freeman 1968).

A key component of male reproductive behavior in many *Bombus* species involves scent-marking patrol circuits during which they deposit pheromones on substrates such as branches, leaves, or stems as they establish regular flight paths to attract receptive queens (Frank 1941; Haas 1946; Haas 1949; Haas 1952; Kullenberg 1973; Harano et al. 2018), a behavior first described by Darwin (Freeman 1968). These patrol routes are typically stable, with males revisiting specific marking sites repeatedly (Frank 1941; Harano et al. 2018). These circuits share some structural resemblance to the traplines used by workers foraging for nectar (Thomson et al. 1997), but may reflect different behavioral priorities and decision-making rules.

Although bee males contribute to pollination and influence mating dynamics (Kraus et al. 2009; Ogilvie and Thomson 2015), their movement strategies remain poorly understood. In contrast to workers, whose foraging patterns and spatial behaviors have been extensively studied (Osborne et al. 2008; Goulson 2010; Woodgate et al. 2016), male movement ecology—particularly how it is shaped by environmental factors such as resource distribution and nectar availability—has received limited attention (Smith et al. 2019; Woodgate et al. 2021; Mola and Williams 2025). Yet understanding male movement is crucial: it determines encounter rates with potential mates (Paxton 2005), mediates pollen dispersal across habitats (Ogilvie and Thomson 2015), and thus influences gene flow and population structure of both bees and plants (Jaffé et al. 2009; Kraus et al. 2009). Quantifying male foraging and reproductive behaviors in controlled spatial environments is therefore essential, not only to illuminate how males navigate ecological tradeoffs, but also to clarify their role in mating systems and pollination.

To address this gap, we used high-resolution 3D video tracking of the spatial movement patterns of male *Bombus terrestris* in a controlled flight cage environment. This allowed us to track individual males with unprecedented accuracy, enabling detailed analysis of their movement decisions over extended periods. The flight cage contained artificial flowers (providing nectar) and scent-marking locations (nonrewarding branches), mimicking the dual-resource environment males encounter in nature. We systematically manipulated the spatial arrangement of these elements and the nectar delivery rate to assess how males navigate tradeoffs between feeding and reproductive efforts. Specifically, we sought to answer 4 key questions: (i) Do males trade off foraging effort for reproductive signaling when nectar availability or spatial resource configuration changes? (ii) What movement strategies do males follow when visiting successive items? (iii) Do they adjust the frequency of revisits over time? (iv) Do they establish repeatable routes, and if so, are these routes affected by spatial configuration and nectar reward rates?

## Methods

### Experimental setup

Experiments were conducted in a flight cage (300 cm L × 300 cm W × 150 cm H) within a controlled laboratory environment between June 2021 and July 2022. The cage was enclosed with white tulle fabric (Handi Stitch, Amazon UK), a fine mesh material with an estimated pore size of c.a. 0.5 mm, suitable for containing bumblebees while allowing airflow and visibility. The cage was surrounded by white curtains to standardize visual cues for the bees and to optimize 3D tracking by providing strong contrast between the bees and the background. Two machine vision cameras (Basler acA1300, Basler AG, Germany) equipped with Kowa 4.4 to 11 mm varifocal lenses (Kowa Company Ltd., Japan) were positioned outside the enclosure and oriented toward the flight volume at an angle of approximately 50° relative to each other. This configuration enabled stereoscopic 3D tracking of bee trajectories. The floor was covered with laminated printouts of red and white Julesz patterns, which are random-dot textures used to provide high spatial frequency contrast without distinct landmarks (Julesz 1962). These patterns enhanced optic flow cues for flight control and improved the accuracy of 3D tracking by providing a high-contrast background against which bees could be reliably detected. A 40 × 41 cm piece of purple-painted cardboard was placed between the camera bases to serve as a spatial reference point for the bees. Environmental conditions were maintained at 21.8 ± 0.02 °C and 46.2 ± 0.3% relative humidity, recorded continuously using a digital sensor.

### Subjects

We used male *B. terrestris* from 10 commercial colonies (each containing a queen and 40 to 50 workers) and from additional male-only boxes (∼50 males per box), all purchased from Agralan Ltd. (Wiltshire, UK). Males in the boxes were almost certainly sourced from multiple colonies, given the finite number of males produced per colony (Vaidya et al. 2018) and the number of males supplied per box. Colonies were housed in wooden nest boxes (28 × 16 × 11 cm) lined with cat litter to regulate moisture levels. A 25% (w/w) sucrose solution was provided via feeders, and nests were provided with Natupol pollen (Koppert, Suffolk, UK) twice weekly.

A total of 62 males were initially included in the experiment. However, 11 were excluded because of escape, persistent flight instability (eg repeated falling), feeder dislodging, incomplete video recordings, or tracking errors, resulting in a final sample size of 51 males.

### Flight cage items

**Figure 1:**
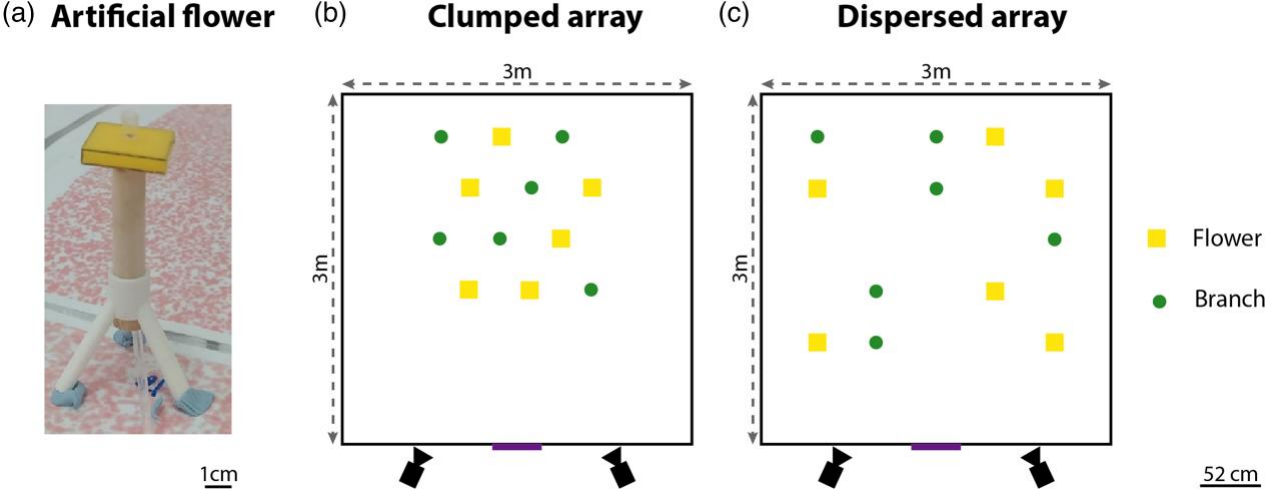
Experimental spatial arrangements and artificial flower. a) Artificial flower: Photograph of an artificial flower, showing the white 3D-printed tripod, vertical wooden support, yellow plastic visual cue, and sucrose feeder tip. b) Clumped array: Branches (circles) and artificial flowers (squares) were arranged in a uniform grid at 52 cm intervals within a 135 × 135 cm area (c.a. 1.82 m^2^), simulating a densely packed patch. c) Dispersed array: Items were placed at irregular distances across a 208 × 208 cm arena (c.a. 4.33 m^2^), using a 5 × 5 grid as a placement guide. Nearest-neighbor distances ranged from 52 cm to c.a. 214 cm, resulting in a more spatially heterogeneous layout. 3D tracking cameras are represented in black and the purple bar represents a central landmark. The flight cage measured 3 × 3 m.

To simulate a realistic environment for foraging, scent-marking, and patrolling, we arranged 6 *Cotoneaster horizontalis* branches and 6 artificial flowers in the flight cage (Fig. 1). *C. horizontalis* is a shrub whose flowers are naturally visited by bumblebees (Corbet and Westgarth-Smith 1992) and was used as a substrate for male scent-marking behavior. Leaf-bearing branches were selected to have minimal lateral branching and measured approximately 15 cm, providing a relatively uniform and accessible structure for landing and marking. Each branch was mounted on a 3D-printed white tripod (10 cm high) to ensure stability and consistent presentation across trials.

The artificial flowers (Fig. 1a) were designed to deliver controlled amounts of nectar substitute (BIOGLUC, Biobest, Belgium), an invert sugar syrup containing glucose, fructose, and sucrose (Fratoni et al. 2020). We used this instead of sugar water as it does not crystallize as quickly, an important consideration given the extended duration of our experiments. Each flower consisted of a white 3D-printed tripod (5 cm high), supporting a 6 cm wooden cylinder (1 cm radius) with tubing (3 mm outer diameter, 1 mm inner diameter) to facilitate nectar flow. A yellow square plastic chip (2.5 × 2.5 × 0.5 cm) served as a visual cue, and an Eppendorf tube tip (0.7 cm deep) held the sucrose solution. The Eppendorf tube depth was selected to match the tongue length of *B. terrestris* males (c.a. 0.7 cm, Gérard et al. 2023), ensuring efficient feeding.

Artificial flowers were automatically refilled every hour using Tempatron AM24 drive motors and a gear kit-controlled cam sequencer system (RS Components, Corby, UK). The refill system was active for 15 min at the start of each hour, followed by a 45-minute pause, repeating this cycle continuously throughout the day. Two different sucrose delivery rates were implemented to simulate conditions of high and low nectar availability. Under the high-rate condition, nectar was delivered at 4.64 ± 0.16 μL/min, resulting in 69.6 ± 0.16 μL per flower over each 15-min refill period. In contrast, under the low-rate condition, nectar was dispensed at 1.63 ± 0.23 μL/min, yielding 24.4 ± 0.23 μL per flower per refill cycle. While these values exceed typical nectar secretion rates of individual flowers in bumblebee-pollinated species such as *C. horizontalis* (c.a. 0.01 to 0.05 μL/min; Dmitruk et al. 2022) and *Digitalis purpurea* (c.a. 0.04 μL/min; Gaffal et al. 1998), they fall within the upper range reported for large, nectar-rich flowers such as *Cucurbita pepo* and *C. maxima* (≈20 to 130 µl h^−1^; Vidal et al. 2006; Dmitruk 2008).

Given that the mean maximum crop fill in *B. terrestris* males is 118.5 ± 45.8 μL (*N* = 20; Wolf and Chittka 2016), bees would need to collect all nectar available in 2 fully replenished high-rate flowers or 5 fully replenished low-rate flowers to fill their crop. This setup thus allowed us to investigate how changes in nectar availability influenced male bee foraging behavior and movement patterns.

### Spatial arrangements of items

Two spatial configurations were tested, representing contrasting resource distributions: a clumped array and a dispersed array (Fig. 1b and c).

In the clumped array, items (branches and artificial flowers) were arranged at regular intervals (52 cm) within a restricted area (135 cm × 135 cm, c.a. 1.82 m^2^) of the larger 3 m × 3 m flight cage. This created a dense patch of habitat within the enclosure that mimicked a tightly packed patch of vegetation.

In the dispersed array, items were placed at irregular distances across a 208 cm × 208 cm area (c.a. 4.33 m^2^), guided by a 5 × 5 placement grid to achieve a heterogenous layout with variable spacing between items. This resulted in a more spatially complex environment, with nearest-neighbor distances ranging from 52 cm to c.a. 214 cm and fewer local neighbors per item.

Note that the total area occupied by the array within the flight cage differed between configurations, and spatial regularity was not controlled independently of area size. Consequently, any behavioral differences observed between the 2 spatial arrangements reflect the combined effects of spacing structure and density.

A total of 51 males were tested across the spatial and nectar availability conditions: 12 in the dispersed array with low nectar availability, 12 in the clumped array with low nectar, 12 in the clumped array with high nectar, and 15 in the dispersed array with high nectar.

### Training procedure

To facilitate learning, 6 males were removed from their colony or male box 1 day before testing and marked with numbered, colored disks (Bienen-Voigt & Warnholz GmbH & Co. KG, DE) glued to their thoraces for individual identification. The bees were then placed in a wooden training box (28 × 16 × 11 cm) containing 6 yellow plastic chips (matching those in the artificial flowers). A drop of BIOGLUC was manually pipetted onto each chip to establish a color-reward association. Refills were provided immediately after depletion, and the bees were observed until they had contacted the sucrose with their proboscis at least once.

### Testing procedure

The 6 male bees were released into the flight cage at c.a. 15:30 on the day prior to testing. The cage operated under a light-dark regime of 8 h of light and 16 h of darkness, with lights on from c.a. 09:00 to 17:00. Following release, bees experienced c.a. 1.5 h of light (from 15:30 to 17:00), during which they began initial exploration and familiarization with the new environment. On the day of testing, males were observed until at least 1 individual had scent-marked a branch, fed from an artificial flower, and demonstrated sustained flight (flower scent-marking was also recorded; see Behavioral classification). Once a suitable bee was identified, individual testing began. Only one bee was tested at a time to eliminate social interference; other males were gently removed from the flight space using bee marking cages (Thorne, UK) and returned to the training box, a procedure that did not visibly disturb the focal individual. Because males were group-housed prior to individual testing, scent marks from multiple individuals may have been present in the arena at the start of trials. The extent to which males avoid or overmark conspecific scent marks was not quantified in this study. The focal bee was then recorded for 6 h, from 09:30 to 15:30, using 2 Basler GenICam cameras for 3D tracking. This consistent 6-h observational window allowed comparison across individuals and conditions while controlling for potential circadian variation.

Males not selected as focal individuals on a given day could later be re-introduced as candidates, but each male was tested as focal only once. All individuals were uniquely identified, allowing us to track any prior candidate use. Occasional re-use of nonfocal candidates was evenly distributed among treatment combinations (χ^2^ = 2.96, *P* = 0.400) and is therefore unlikely to have introduced bias.

### Data analysis

We used the software package Track3D (Noldus Information Technology, Wageningen, The Netherlands) as an add-on to EthoVision XT 16.0.1538 to generate 3-dimensional tracking data. EthoVision XT extracted 2D coordinates from 2 synchronized Basler video cameras, and Track3D combined these into a set of 3D coordinates (X, Y, Z) using stereo triangulation. This reconstruction was based on prior calibration with CentroidFinder, which aligned the image coordinates to real-world spatial dimensions. The cameras recorded bee positions at 30 frames per second.

#### Behavioral classification

Behavioral modes were classified using a simple hand-crafted algorithm using a small number of thresholds for movement statistics. These thresholds were initially informed by exploratory analyses and comparison to expert-labelled segments, but the final classification rules were selected to approximate expert observations as objectively and consistently as possible. We validated behavioral classifications against manual scoring on 4 independent individuals (Tables S1 and S2) and performed ±20% threshold-sensitivity analyses, reclassifying all segments and re-running statistical models under perturbed thresholds. Both analyses confirmed robust classifications and conclusions (Figures S1–S3, Table S3). Three distinct behavioral modes were analyzed: Feeding, Scent-Marking, and Patrolling.

First, the 3D positional data for each track were broken into 0.5 s segments and movement statistics calculated for each segment: speed, turning angle, distance traveled, and proximity to the nearest flower or branch. Behavioral classification was hierarchical rather than based on mutually exclusive speed categories: segments were first classified as flight or nonflight, and specific behaviors were then identified within these broader classes using additional criteria. Segments in which the mean movement speed exceeded 7 cm/s were classified as flight, using a deliberately low threshold to capture all airborne movement rather than to distinguish among flight behaviors. It was not possible to use movement speed to differentiate between walking and sitting still because bees often move very slowly. Thresholds for angular momentum and distance moved can be effective at differentiating different walking and sitting behaviors but since the behaviors we were interested in (feeding and scent-marking) can involve a range of movement speeds we treated all walking and sitting movement as the same for the purposes of further behavioral classification.

Feeding was defined as any instance in which a bee was recorded walking or sitting within 20 cm of a flower and no more than 5.5 cm above or below the flower platform. This spatial threshold was used because the resolution and distance of the cameras did not allow for direct visual confirmation of feeding, and slight positional variation of flower placement occurred between trials despite marked floor guides. The 20 cm radius accounts for this variability while ensuring a consistent, conservative estimate of flower visits likely associated with feeding. To ensure interactions were specific to the flower platform, body movement had to be predominantly horizontal, with a horizontal-to-vertical movement ratio exceeding 1.5.

Scent-Marking was characterized by a bee walking or sitting within a 20 cm radius of an item at a height of no more than 1 cm above it, where vertical body movement was predominant (horizontal-to-vertical movement ratio below 1). In the context of this study, bees exhibited such behavior on both artificial flowers and branches.

We used the term Patrolling to describe flight behavior near flowers or branches involving slow, highly maneuverable movement, consistent with environmental inspection in search of mates. Although similar movement patterns can occur during local search for food, the behavior observed here is consistent with patrolling (mate-searching) flights described in male bees across multiple taxa (Alcock et al. 1978; Eickwort and Ginsberg 1980; Paxton 2005). In many species, males traverse and repeatedly visit locations while searching for receptive females, often independently of floral resource use, and may deposit scent marks along these routes (Ayasse et al. 2001). Consistent with this, patrolling events in our study were frequently observed around nonrewarding substrates and were not systematically followed by feeding, supporting their interpretation as reproductive behavior. However, as movement patterns alone cannot fully distinguish behavioral motivation, some overlap with foraging-related inspection cannot be excluded. A patrolling bout began when a bee entered a 20 cm radius around a flower or branch and flew no more than 10 cm above it, while maintaining a flight speed below 50 cm/s (a conservative threshold for slow, looping flight) and an angular velocity (ie, rate of change in heading direction) above 125°/s. These thresholds were applied only to segments already classified as flight and were chosen to distinguish patrolling from faster, straighter transit flights, capturing the characteristic tight turning and hovering-like motion typical of inspection behavior. A patrolling event was considered to have ended when the bee: (i) landed on a branch, the cage floor, or netting; (ii) ascended more than 45 cm above the level of the items (suggesting transition to exploratory flight); or (iii) went more than 5 seconds without meeting the criteria for further inspections.

#### Hypothesis testing

All statistical analyses were conducted in R (v.4.4.2; R Core Team 2024), using the lme4 (Bates et al. 2015), emmeans (Lenth 2024), and performance (Lüdecke et al. 2021) packages. We used linear mixed-effects models (LMMs) to test how male behavior varied in response to spatial resource distribution (clumped vs. dispersed), nectar availability (low vs. high), and behavior type (feeding, scent-marking, patrolling). All models included Male ID as a random intercept. Significance of fixed effects was assessed using Type III Wald χ^2^ tests (car package; Fox and Weisberg 2019), and estimated marginal means were extracted using emmeans with Tukey correction for multiple comparisons. Where applicable, predicted values were back-transformed from log-transformed response variables. Model fit was assessed using marginal and conditional R^2^ (performance package). Model selection was based on Akaike's Information Criterion (AIC), with candidate models derived from a global model including all main effects and interactions. Models within ΔAIC ≤ 2 were considered to have equivalent support, and the most parsimonious model was selected. As a result, the retained interaction structure differed slightly among response variables, with only those interactions improving model fit (ΔAIC ≤ 2) included in each final model. Full model selection tables, including model structure, number of parameters (k), AIC values, ΔAIC, and AIC weights, are provided in the Supplementary material. A minimum duration threshold of 0.5 s was applied to all behavioral events (frequencies, total duration, and per-event duration) to exclude transient detection artifacts while retaining brief but genuine behavioral interactions, as validated through manual video scoring (minimum observed feeding duration: 0.533 s).

Behavioral frequency (log-transformed) was modeled as a function of behavior type, spatial resource distribution, nectar availability, and their interaction (behavior × spatial distribution). Marginal *R*^2^ = 0.168; Conditional *R*^2^ = 0.642.

Total behavioral duration (log-transformed) was modeled with fixed effects of behavior type, nectar availability, spatial resource distribution, and the interactions behavior × spatial distribution and nectar availability × spatial distribution. Marginal *R*^2^ = 0.188; Conditional *R*^2^ = 0.522.

Per-event duration (log-transformed) was modeled as a function of behavior type, spatial resource distribution, nectar availability, and their interactions (behavior × spatial distribution, nectar availability × spatial distribution). Due to the large sample size, post hoc comparisons used asymptotic approximations. Marginal *R*^2^ = 0.489; Conditional *R*^2^ = 0.523.

To analyze transition patterns in movement, we examined pairwise transitions between consecutive visits, calculating for each transition both the Euclidean distance and the heading of the displacement vector between visited locations in the x–y plane using Python (version 3.11.7). Heading was computed from the coordinates of successive visited items using the function arctan2(Δy, Δx), converted to degrees, and folded into the range 0 to 180° to remove directional bias (left vs. right). Specifically, negative angles were converted to positive values, and angles >180° were transformed as 360° − θ. This metric therefore represents the orientation of each transition relative to the arena *x*-axis. The Mann–Whitney U test was used to compare distributions, and Cohen's d was computed as an effect size measure to quantify differences. Return cycles were quantified as the number of intervening visits between successive visits to the same location within a time window. For each individual and time block, we calculated the sequence of visited locations and computed the number of visits occurring between repeated visits to each location (ie revisit intervals). The mean and variance of these revisit intervals were then calculated for each time window and used as response variables. Return-cycle metrics were analysed in 2 complementary ways. Mean return cycle was analysed using LMMs, with Male ID as a random intercept and nectar availability, array configuration, time, and their interactions included as fixed effects. Return-cycle variance, which was zero-inflated and strongly right-skewed, was analysed using a generalized linear mixed model with a Tweedie distribution and log link (glmmTMB; Brooks et al. 2017), again including Male ID as a random intercept. Immediate revisits were also modeled using a Negative Binomial distribution, including Male ID as a random effect, along with visit block, array configuration and behavior type as interaction terms. Here, an immediate revisit was defined as 2 consecutive visits to the same location (eg, A → A) without an intervening visit elsewhere. This applied equally to feeding, scent-marking, and patrolling behaviors.

To assess whether male bees used repeated routes between items, we calculated the Determinism Index (DET) following Ayers et al. (2015). This index quantifies sequence repetition, where values closer to 1 indicate highly deterministic movement patterns, while values near 0 indicate random movement. DET was calculated using sequences ranging from 3 to 5 items in length. To evaluate whether movement patterns resulted from structured decision-making rather than randomness, DET values (excluding immediate revisits) were compared with those generated by movement models simulating specific navigation rules within the spatial array, as well as to the observed movement sequences of the focal individuals (hereafter “Real Bees”). We implemented 4 movement models to compare against observed bee behavior: Nearest Neighbor (NN): Bees moved to the closest item; Direct Neighbor (DN): Bees moved to nearby, unoccluded items within 2× the nearest-neighbor distance and outside a 25° masking angle, representing a broader but vision-constrained local choice; Low Turn (LT): Bees moved in directions that minimized turning angle, mimicking persistence in directionality; Random (R): At each step, the next item was selected uniformly at random from all available items, without applying spatial or directional constraints. This model represents nondirected exploration in the absence of spatial decision rules. For all models, movement sequences were simulated independently as discrete sequences of 50 transitions, and 20 replicate sequences were generated per condition. These models were implemented from scratch in Python using geometric and probabilistic rules. Normality of DET values was assessed using Shapiro-Wilk tests. Differences among models (NN, DN, LT, Random, and Real Bees) were evaluated using Kruskal–Wallis tests, followed by Dunn's post hoc tests with Bonferroni correction for multiple comparisons.

## Results

### Behaviors

Male bumblebee behavior varied in response to the spatial distribution of resources, with additional effects of nectar availability observed in specific contexts. These effects were primarily evident in feeding behavior, whereas reproductive signaling behaviors (patrolling and scent-marking) remained comparatively stable.

#### Behavioral frequencies

**Figure 2:**
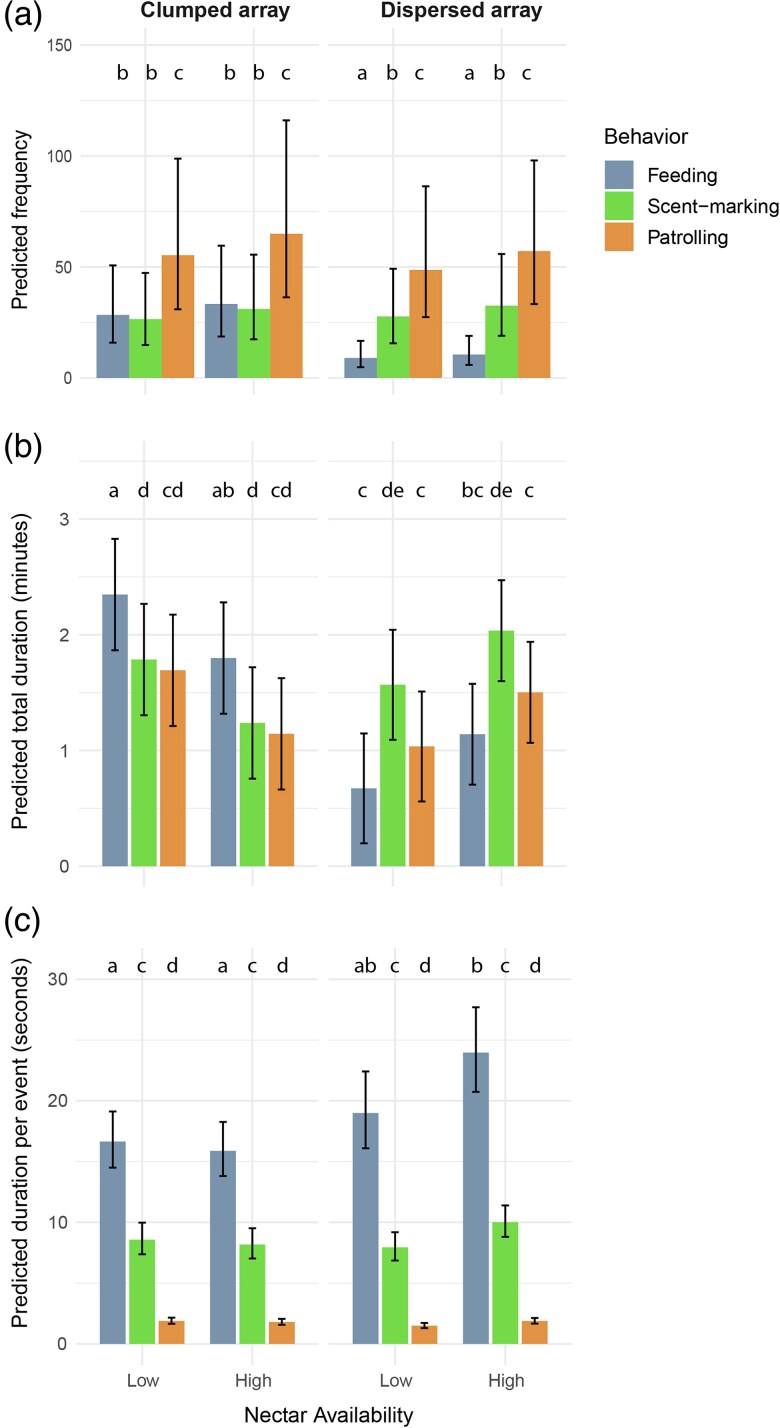
Predicted behavioral metrics for male bumblebees in relation to spatial resource distribution and nectar availability. a) Predicted frequency of behaviors from a linear mixed model, expressed as the total number of behavioral events per individual over the 6-h observation period. b) Predicted total duration of each behavior (in minutes), summed across the same 6-h observation period. c) Predicted per-event duration of each behavior (in seconds). All values are back-transformed from log-transformed data. Error bars represent 95% confidence intervals based on estimated marginal means. All models included Male ID as a random effect. Treatments are shown across nectar availability (low vs. high) and spatial distribution (clumped vs. dispersed). Behavior types are color-coded and separated by facets in panels a–c). A total of 51 males were tested: 12 in the dispersed–low nectar condition, 12 in clumped–low, 12 in clumped–high, and 15 in dispersed–high. Letters denote Tukey-adjusted pairwise differences. Groups sharing a letter do not differ.

We examined whether male bumblebee behavior differed in frequency across spatial resource distribution (clumped vs. dispersed), nectar availability (low vs. high), and behavior type (feeding, scent-marking, patrolling). Behavioral frequency represents the total number of behavioral events recorded per individual during the standardized 6-h observation period (Fig. 2a).

A linear mixed model revealed a significant interaction between behavior type and spatial resource distribution (χ^2^(2) = 13.80, *P* = 0.001), indicating that behavioral frequencies varied depending on spatial arrangement. Main effects of behavior type (χ^2^(2) = 12.14, *P* = 0.002) and spatial resource distribution (χ^2^(1) = 9.90, *P* = 0.002) were also significant, reflecting overall differences in behavior frequency across conditions. Nectar availability had no significant effect (χ^2^(1) = 0.30, *P* = 0.584).

Tukey-adjusted post hoc comparisons of estimated marginal means revealed that in the clumped array, patrolling occurred more frequently than both feeding (estimate = −0.67, SE = 0.23, *P* = 0.015) and scent-marking (estimate = 0.74, SE = 0.23, *P* = 0.006), while feeding and scent-marking did not differ (*P* = 0.952). In the dispersed array, patrolling again occurred more frequently than both feeding (estimate = −1.69, SE = 0.25, *P* < 0.001) and scent-marking (estimate = 0.56, SE = 0.22, *P* = 0.033), and scent-marking occurred more frequently than feeding (estimate = −1.13, SE = 0.25, *P* < 0.001).

Within-behavior comparisons showed that feeding frequency was significantly reduced in the dispersed array compared with the clumped array (estimate = 1.15, SE = 0.37, *P* = 0.002), while no spatial differences were observed for patrolling or scent-marking (*P* > 0.712).

#### Total behavioral duration

We tested whether the total time spent performing each behavior varied with spatial resource distribution, nectar availability, and behavior type (Fig. 2b).

A linear mixed model revealed a significant interaction between behavior type and spatial resource distribution (χ^2^(2) = 27.62, *P* < 0.001), as well as an interaction between nectar availability and spatial distribution (χ^2^(1) = 6.18, *P* = 0.013). Main effects of behavior (χ^2^(2) = 11.84, *P* = 0.003) and spatial distribution (χ^2^(1) = 4.06, *P* = 0.044) were also significant. Nectar availability had no significant main effect (χ^2^(1) = 3.41, *P* = 0.065).

Tukey-adjusted post hoc comparisons showed that for feeding, total duration was significantly lower in the dispersed–low condition compared with both clumped–low (*P* < 0.001) and clumped–high (*P* = 0.007), but did not differ significantly from the dispersed–high condition (*P* = 0.352).

For patrolling and scent-marking, no significant differences in total duration were detected between conditions (*P* > 0.076), suggesting consistent investment in these behaviors regardless of environmental variation.

#### Per-event behavioral duration

We tested whether males adjusted the duration of individual behavioral events in response to changes in spatial resource distribution, nectar availability, and behavior type (Fig. 2c).

A linear mixed model revealed significant effects of behavior type (χ^2^(2) = 5,501.16, *P* < 0.001), as well as interactions between behavior and spatial resource distribution (χ^2^(2) = 43.52, *P* < 0.001) and between spatial distribution and nectar availability (χ^2^(1) = 4.38, *P* = 0.036). The main effects of nectar availability and spatial distribution were not significant (*P* > 0.230).

Tukey-adjusted post hoc comparisons showed that feeding events lasted significantly longer than both scent-marking and patrolling events in all treatment combinations (*P* < 0.001). For feeding, per-event duration was longest in the dispersed–high condition, which was significantly longer than in both clumped–high (*P* < 0.001) and clumped–low (*P* = 0.002). However, feeding durations in the dispersed–high and dispersed–low conditions did not differ significantly (*P* = 0.360).

Per-event durations for scent-marking and patrolling did not differ significantly across conditions (*P* > 0.060), indicating that the temporal structure of reproductive signaling was stable across environmental treatments.

### Movement strategies

**Figure 3:**
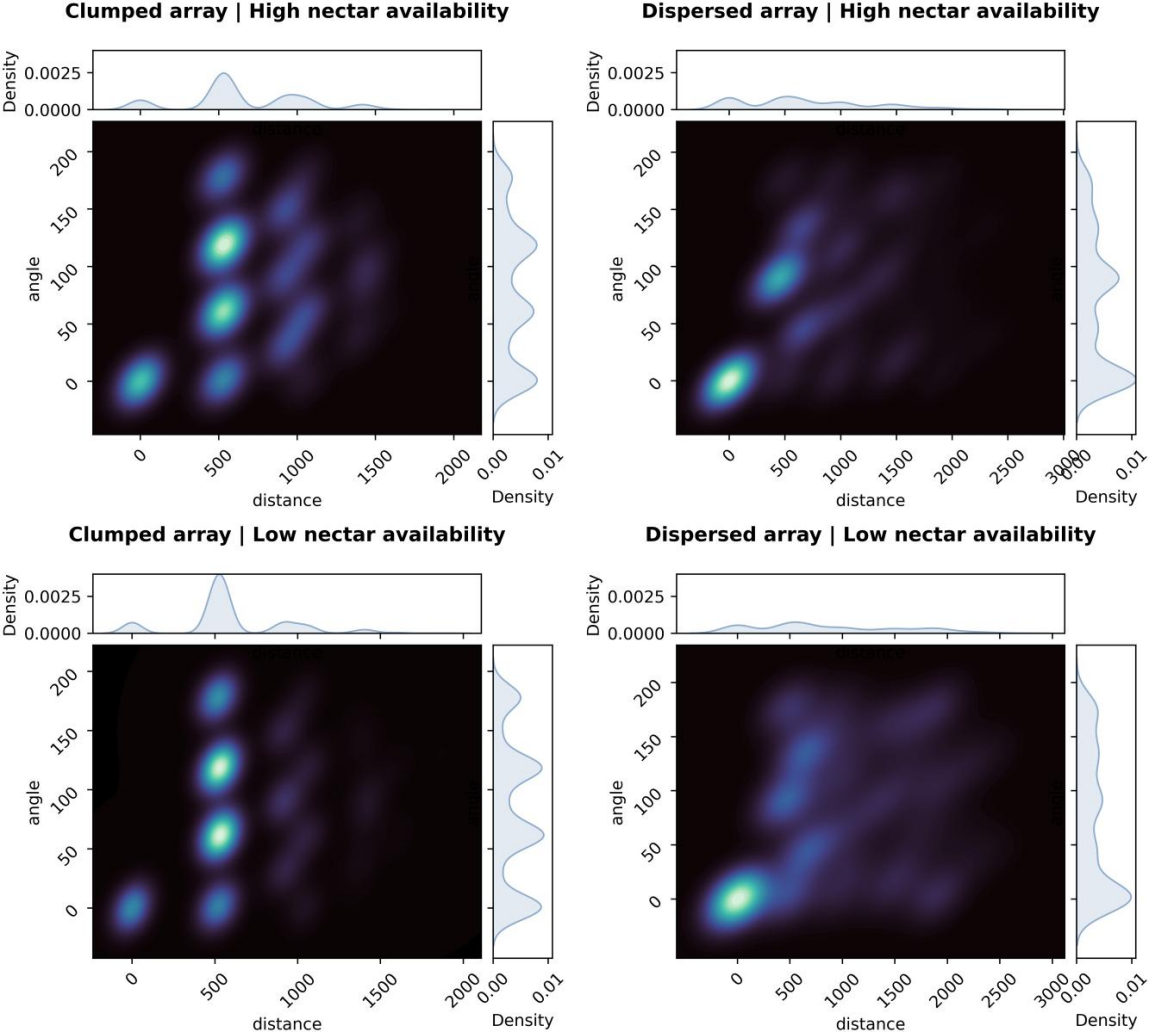
Density plots of movement transitions between consecutive visits to items (artificial flowers or branches), shown as a function of distance and direction (angle), across spatial arrays and nectar availability conditions. Each panel represents 1 of 4 experimental treatments: clumped (left) or dispersed (right) spatial arrays under high (top) or low (bottom) nectar availability. The *x*-axis indicates the distance (mm) between successive visits, and the *y*-axis shows the movement heading (°), defined as the orientation of the displacement vector between consecutive visits relative to the arena's *x*-axis (0° = forward, 90° = lateral, 180° = backward). Brighter areas represent higher densities of transitions. Marginal density plots show the distribution of distances and angles separately. Angles and distances are continuous; banding reflects the discrete geometry of fixed item positions.

To examine how nectar availability and spatial configuration influenced movement behavior, we analyzed pairwise transitions between consecutive item visits, focusing on the distance and heading (orientation relative to the arena x-axis) of each movement. These transition metrics allowed us to quantify how bees adjusted their foraging strategy across the 4 experimental conditions (Fig. 3).

Across both spatial configurations, males tended to visit closer locations when nectar availability was low, consistent with a more localized foraging strategy under limited resources. In the clumped array, individuals moved significantly shorter distances at low nectar availability compared with high (*U* = 8,163,133, *P* < 0.001, *f*^2^ = 0.18). A similar pattern was observed in the dispersed array, where males also reduced movement distances under low nectar availability (*U* = 2,259,686, *P* < 0.001, *f*^2^ = 0.25). By contrast, when nectar was abundant, individuals traveled farther, likely reflecting a more exploratory strategy aimed at maximizing intake from replenished sources.

Directionality of movement also varied with nectar availability, particularly in the dispersed array. Males exhibited more consistent directional movements when nectar availability was low (*U* = 2,490,456, *P* = 0.019, *f*^2^ = 0.05), although the effect size was small. Taken together, shorter movement distances and more consistent directionality under low nectar conditions suggest a more localized movement strategy, whereas longer and more variable movements under high nectar conditions suggest broader exploration.

### Revisitation patterns and movement optimization

**Figure 4:**
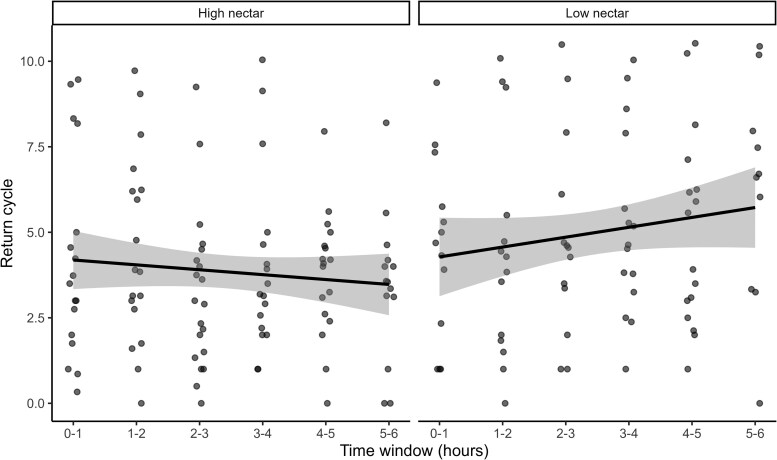
Return cycles across successive time windows under high and low nectar availability. Points represent individual observations, and lines indicate fitted linear trends (± SE).

To determine whether males progressively optimized their movement patterns, we analysed return cycles during patrolling and scent-marking over time (Fig. 4). If males were developing more structured visitation sequences, we would expect an increase in return cycles over time, reflecting longer intervals between successive visits to the same locations. However, return cycles did not show a consistent increase over time across conditions (Fig. 4), as revisit spacing increased under low nectar availability but remained unchanged under high nectar availability.

Return cycles did not change significantly over time overall (*β* = −0.25 ± 0.19 SE, *t* = −1.30, *P* = 0.195). However, there was a significant interaction between time and nectar availability (*β* = 0.80 ± 0.29 SE, *P* = 0.006). Post hoc analyses revealed that return cycles increased over time under low nectar conditions (*β* = 0.55 ± 0.22 SE, *P* = 0.012), whereas no significant change was observed under high nectar conditions (*β* = −0.25 ± 0.19 SE, *P* = 0.195). Return-cycle variability did not decrease over time. Although there was a tendency for variability to increase under low nectar conditions (*β* = 0.30 ± 0.16 SE, *z* = 1.87, *P* = 0.062), the interaction did not reach conventional significance levels.

**Figure 5:**
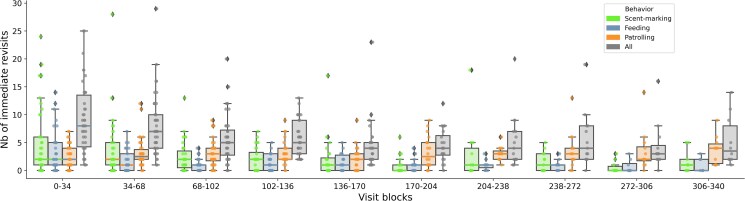
Effect of experience on immediate revisits during scent-marking, feeding, and patrolling. Boxplots show the number of immediate revisits (2 consecutive visits to the same location, regardless of behavior type) per block of 34 visits, allowing assessment of changes in revisitation patterns over time. Scent-marking, feeding, and patrolling are represented separately. Boxplots display medians, quartiles, and 10th to 90th percentiles (whiskers), while dots indicate individual data points.

To further examine how experience influences immediate revisitation patterns, we analyzed the number of immediate scent-marking, feeding, and patrolling revisits across blocks of visits over time (Fig. 5). Here, an immediate revisit was defined as a return to the same location without visiting any other item in between. Males performed fewer immediate scent-marking revisits as they gained experience with the array, suggesting they were less likely to return to the same marking locations in quick succession (*β* = −0.73, SE = 0.10, *z* = −7.45, *P* < 0.001). Similarly, immediate feeding revisits declined significantly over time, likely as a response to nectar depletion, prompting males to seek alternative locations (*β* = −0.35, SE = 0.09, *z* = −3.92, *P* < 0.001). In contrast, immediate patrolling revisits remained stable, indicating that males did not alter their patrolling revisitation patterns with experience (*β* = 0.06, SE = 0.06, *z* = 0.96, *P* = 0.336).

### Route repetition

**Figure 6:**
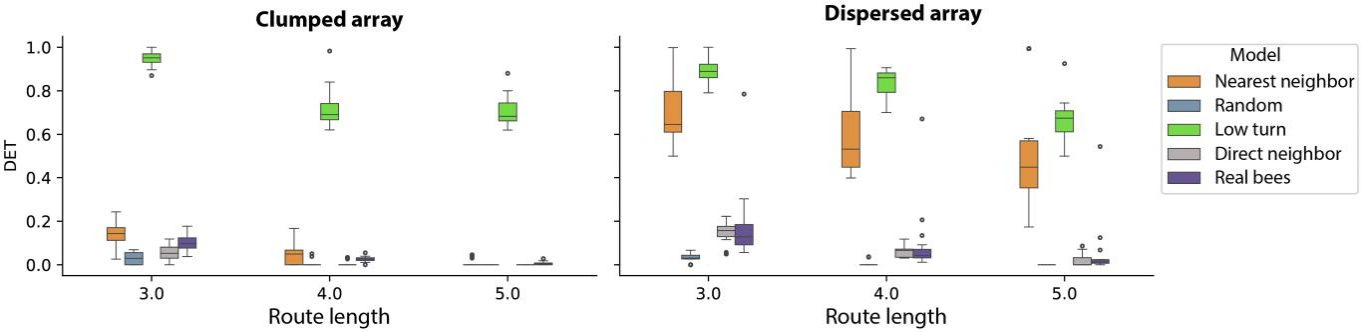
Determinism Index of male bumblebee item visitation sequences in clumped and dispersed arrays, compared with simulated movement models. Boxplots show DET values for real bees and simulated models (NN, random, LT, and DN) across 3-, 4-, and 5-visit sequences.

To assess structured revisitation patterns, we calculated values of Determinism Index (DET) derived from bee movements and compared them to simulated movement models implementing rigid rules of thumb (Fig. 6).

In the clumped array, males tended to move to the closest or visually accessible nearby item, with DET values not significantly different from the nearest-neighbor or the direct-neighbor models (Dunn test after Kruskal–Wallis, NN: *P* = 1.000, DN: *P* = 0.145). However, bumblebee movements significantly differed from the random and low-turn models (*P* < 0.001).

In the dispersed array, males followed a direct-neighbor movement pattern, with DET values not significantly different from the direct-neighbor model (Dunn test after Kruskal–Wallis, *P* = 1.000). In contrast, DET values differed significantly from the nearest-neighbor, random and low-turn models (*P* < 0.001).

## Discussion

This study provides new insights into the movement strategies of bumblebee males, showing how spatial resource configuration and nectar availability jointly shape foraging and reproductive behaviors. Unlike bumblebee workers, which tend to optimize their foraging routes for colony provisioning (Ohashi et al. 2007; Lihoreau et al. 2012; Woodgate et al. 2017), males must independently balance energy intake with mate-seeking, leading to movement patterns associated to distinct behavioral tradeoffs. Resource distribution had a strong influence on feeding behavior, while scent-marking and patrolling remained consistent, suggesting a prioritization of reproductive signaling over energetic optimization. Nectar availability, somewhat unexpectedly, had minimal effects on these behavioral tradeoffs, indicating that mate-seeking investment is maintained even when nectar is scarce. While prior studies have described repeated visitation patterns and individual patrol circuits in males (Frank 1941; Harano et al. 2018), we found no strong evidence of systematic route formation within trials, possibly reflecting the limited spatial scale of the experimental environment.

### Males maintained reproductive signaling by adjusting foraging behavior

A key question in male foraging ecology is whether individuals reduce feeding effort to sustain investment in reproductive signaling under varying resource conditions. Our results suggest that males do not reduce signaling behaviors but instead adjust their foraging effort to accommodate energetic constraints. Specifically, scent-marking and patrolling rates were not significantly different across nectar availability and spatial configuration treatments, suggesting that males maintained mate-seeking behavior even when the energetic cost of foraging increased.

The dispersed–low nectar condition imposed the highest foraging cost, as reflected by the lowest feeding frequency and total feeding duration. Under these constrained conditions, males did not reduce reproductive investment but instead fed less frequently and for shorter total durations, while extending the duration of individual feeding events when nectar availability allowed. This pattern indicates a context-dependent shift in foraging behavior that enables males to conserve energy while maintaining consistent signaling effort. Notably, flowers were only refilled once per hour, making it unlikely that longer visits were attempts to wait for replenishment. Instead, longer feeding durations likely reflect more thorough exploitation of the nectar available at the time of arrival. Supporting this, nectar was often observed remaining in the artificial flowers at the end of the day, suggesting that males did not always fully deplete nectar during shorter visits. Rather than a symmetrical tradeoff between foraging and reproduction, the data points to a unidirectional adjustment, in which foraging is modulated to preserve reproductive goals.

Revisitation behavior also varied by behavioral context. Scent-marking and feeding revisits declined over time, likely due to pheromone accumulation deposited by the focal male and nectar depletion. However, because males were group-housed prior to testing, additional marks from other individuals may also have been present and could contribute to reduced revisitation. As responses to conspecific marks were not quantified, the relative importance of self- versus conspecific marking cannot be determined. In contrast, patrolling revisits remained stable, underscoring the persistent nature of reproductive effort. Although similar movement patterns can occur during local search for food, the behavior observed here is consistent with patrolling (mate-searching) flights described in male bees across multiple taxa (Alcock et al. 1978; Eickwort and Ginsberg 1980; Paxton 2005). In our study, these events were frequently observed around nonrewarding substrates and were not systematically followed by feeding, supporting their interpretation as reproductive behavior. However, as movement patterns alone cannot fully distinguish behavioral motivation, some overlap with foraging-related inspection cannot be excluded.

It is important to note that this behavioral stability was observed within the range of nectar conditions tested here. While our nectar delivery rates exceeded average secretion rates for individual wildflowers, they fall within the upper range observed in highly productive species such as *Cucurbita pepo* and *C. maxima* (Vidal et al. 2006; Dmitruk 2008). As such, they represent ecologically relevant conditions that bees may encounter in rich environments. However, under more severe nectar limitation than tested here, males may eventually prioritize feeding over reproductive investment. Future work should explore these lower bounds to determine the robustness and limits of reproductive signaling in *B. terrestris*.

The absence of potential mates or male competitors in our experimental setup likely constrained the behavioral repertoire observed. In natural conditions, males often patrol along shared circuits where they encounter rivals or receptive queens, and such social context can markedly alter signaling dynamics (Paxton 2005; Harano et al. 2018). The presence of competitors may intensify scent-marking and patrolling through increased mate-search effort or territorial interactions, while direct exposure to queens could reduce foraging and increase stationary courtship or hovering behavior near marking sites (Kullenberg 1973; Free 1982). Conversely, isolated males, such as those tested here, may express a baseline level of reproductive investment that reflects intrinsic motivation rather than socially modulated effort. Quantifying the effect of conspecific cues in future experiments would therefore be critical to assess how energetic tradeoffs shift under ecologically realistic mating scenarios.

### Spatial heterogeneity and nectar scarcity triggered conservative movement strategies

Male bumblebees adapted their foraging and movement strategies to environmental constraints shaped by both spatial configuration and nectar availability. In the clumped array, which was spatially homogeneous with evenly spaced and closely situated resources, males predominantly visited items spaced at ∼52 cm, the nearest-neighbor distance, enabling efficient, localized movement. Consistently, route determinism (DET) in this environment did not differ from either the nearest- or the direct-neighbor models, indicating that simple local decision rules based on proximity and visual access can fully account for the observed sequence structure. In contrast, the dispersed array was spatially heterogeneous, with greater variability in inter-item distances and irregular layout. These conditions posed greater energetic challenges. Males in this array fed less frequently and for shorter total durations, especially when nectar was scarce and movement data showed that under low nectar availability males exhibited shorter transitions. Under low nectar availability, males also exhibited increased return cycles, indicating longer intervals between revisits and a tendency to reuse subsets of locations rather than expanding their search. Here, real trajectories were less deterministic than predicted by the nearest-neighbor model but matched the direct-neighbor model, suggesting that when resources are widely spaced, males rely on visually guided rather than purely proximity-based rules to maintain efficiency. This pattern is consistent with an energy-conserving foraging strategy, in which males constrained their activity rather than initiating new exploratory searches.

When nectar availability was high, males in both arrays broadened their foraging range, showing longer and more variable movement transitions—suggesting a shift toward exploratory behavior aimed at maximizing intake from replenished sources. However, this behavioral flexibility does not align with classical optimal foraging models (Pyke 1984), which predict increased movement when local resource intake is low. Instead, males appeared to prioritize movement efficiency under poor conditions, minimizing travel distances. This strategy may limit overall nectar intake but likely serves to conserve energy when foraging is most costly.

These findings build on earlier work by Cartar and Real (1997), who showed that worker bumblebees forage more efficiently in spatially structured environments. Our study extends this pattern to male bumblebees, demonstrating that even under different behavioral goals, males benefit from spatial homogeneity through more economical movement and access to resources. Comparable effects of spatial structure on male movement have also been reported in other taxa, including other insects, where resource distribution shapes male mating dispersion (Wickman and Rutowski 1999), and hummingbirds, where foraging routes between flowers are strongly structured by patchiness (Wolf and Hainsworth 1990). Together, these parallels suggest that spatial heterogeneity imposes a common set of constraints across taxa, with male foragers consistently modifying movement to balance efficiency with reproductive opportunities.

### No evidence of traplining or long-term patrol routes in males

One of the most striking results of this study is the absence of convergence toward stable or repeatable routing strategies in male bumblebees. While movement sequences were structured, they did not become more consistent or predictable over time. While return cycles showed context-dependent temporal changes, with increases under low nectar availability, variance in return cycles did not decrease over time, indicating that movement patterns did not become more regular or predictable. Instead, males followed flexible movement patterns constrained by simple local decision rules, frequently revisiting some sites before fully exploring all available ones. This contrasts with trapline foraging observed in workers (Saleh and Chittka 2007; Ohashi and Thomson 2009; Lihoreau et al. 2012; Lihoreau et al. 2013; Reynolds et al. 2013), and more broadly with predictions from movement ecology that animals should optimize their routes to improve efficiency (Berger-Tal and Bar-David 2015).

This difference may reflect both motivational context and spatial scale. Unlike workers, males do not forage to provision the colony, and thus face less pressure to develop efficient, repeatable routes. Their primary selective pressure is reproductive success, which favors sustained signaling over energetic optimization. While spatial scale may also play a role, our experiment was conducted in a relatively small 3 × 3 m flight cage, where movement costs were low and all sites were accessible with minimal effort. Although traplining has been documented in indoor studies, these typically involved larger arenas (eg 5 to 7 m; Ohashi et al. 2007; Lihoreau et al. 2010; Buatois and Lihoreau 2016; Buatois et al. 2024), suggesting that both spatial limitations and behavioral priorities contributed to the absence of route learning in our study. Studies comparing bee behavior in similar arrays of flowers at multiple spatial scales show that bees develop more stable and optimal traplines as distance between flowers increases (Buatois and Lihoreau 2016).

Early observations described species-typical patrol circuits in male bumblebees (Frank 1941; Haas 1946; Haas 1949). While we observed structured movement patterns in both clumped and dispersed arrays (as indicated by DET values not measurably different from nearest- or direct-neighbor models), these patterns are best explained by simple local decision rules rather than the formation of learned, repeatable routes. Consistent with this, we found no evidence of long-term route fidelity or decreasing revisit variance. The discrepancy with earlier observations likely reflects differences in spatial resolution, scale, and ecological context. Future work using harmonic radar (Riley et al. 1996) or other fine-scale tracking in larger scale field conditions could help determine whether male patrol routes become more structured at larger spatial scales.

Beyond bumblebees, understanding male pollinator movement has broader implications: males of several bee species (eg *Bombus*, *Osmia*, *Habropoda*) have been shown to pollinate effectively despite lacking specialized pollen-collecting structures (Pascarella 2010; Fliszkiewicz et al. 2011; Ogilvie and Thomson 2015; Roswell et al. 2019). Their high mobility, including long-distance mate-searching flights (Kraus et al. 2009) and nocturnal foraging observed in some tropical *Bombus* species (Hsieh et al. 2023), suggests that flexible male movement may sustain pollen transfer when female foraging declines or ceases. Male and female bees also exhibit distinct floral preferences, which could further differentiate their ecological roles in plant-pollinator networks (Roswell et al. 2019). More broadly, these cases reflect a shared dynamic across diverse taxa: male pollinators often move through floral environments in pursuit of mates or sensory stimuli rather than resources yet still effect pollination. Similar patterns are seen in male orchid bees and hoverflies, whose nonforaging floral visitation results in effective pollen transfer (Williams and Whitten 1983; Stökl et al. 2011). These findings underscore the need to account for male pollinator behavior in conservation and monitoring frameworks, not only for their contributions to pollination services and species interactions, but also for their role in sustaining gene flow and effective population size for both bee and plant species across fragmented landscapes (Kraus et al. 2009; Roswell et al. 2019).

## Conclusion and future directions

This study offers novel insights into the movement ecology of male bumblebees, showing that they maintain consistent reproductive signaling while flexibly adjusting their foraging strategies to environmental structure. Rather than converging toward stable movement paths over time, males appear to adopt a responsive, opportunistic strategy—reducing search effort when necessary and extending feeding durations to conserve energy.

Unlike many other species where males scale reproductive investment to energy reserves (Meuche and Grafe 2009; Billings et al. 2019; Dougherty 2021; Fernlund Isaksson et al. 2022), male *B. terrestris* maintain signaling effort even under energetically demanding conditions. This likely reflects strong evolutionary pressure to sustain mate-seeking behaviors despite environmental variability. Instead of cutting back on signaling, males appear to adjust the flexible component of their behavioral budget: foraging. They do so through compensatory strategies, such as increasing feeding duration when nectar is found. In this way, males conserve energy without compromising access to mating opportunities, suggesting that reproductive signaling in this species is behaviorally robust, while foraging effort is the more adaptable component.

Future research should examine whether this behavioral flexibility affects mating efficiency or pollination success, particularly in fragmented or resource-poor landscapes. As landscape structure alters floral distributions, understanding how males navigate complex environments is critical to predicting their ecological roles and reproductive outcomes. Large-scale field studies using tracking systems to record individual flight paths would be especially valuable to assess whether route optimization emerges at broader spatial scales—or whether flexibility remains the dominant strategy in male movement ecology.

## Supplementary Material

arag054_Supplementary_Data

## Data Availability

Analyses reported in this article can be reproduced using the data provided by (Rossi 2026).
