# Supplementary material for: Male bumblebees sustain mate-seeking by adjusting foraging to environmental conditions
Source: Behav Ecol. 2026 May 19;37(4):arag054. doi: 10.1093/beheco/arag054 (PMC13247596; doi:10.1093/beheco/arag054)
Supplement: arag054_Supplementary_Data [file arag054_supplementary_data.docx]

**Supplementary Methods:**

**Validation of automated behavioral classification using manual scoring**

Manual behavioral scoring was conducted by the first author (N.R.), who was not involved in the development of the automated behavioral classification algorithm and was blind to the automated classifications at the time of manual scoring. Scoring was performed on raw two-dimensional video recordings from one of the two synchronized cameras.

Frame-by-frame comparison between manual scoring and automated classification was not possible because three-dimensional trajectories are generated only during periods when the bee is simultaneously detected in both camera views. When the bee is occluded, leaves the shared field of view, or is detected by only one camera, no three-dimensional position is reconstructed, resulting in discontinuous tracking data. Consequently, automated classifications do not exist for every video frame, preventing continuous temporal alignment with manually scored video footage.

Four individual males (IDs 10, 18, 39, and 41), representing all experimental treatments and not used during algorithm development, were selected for validation. For each individual, approximately 30 minutes of continuous video were manually annotated.

Behavioral scoring was event-based and focused on visits to items (artificial flowers or branches), rather than on frame-level time budgets. Feeding was scored when a male landed on an artificial flower and remained relatively stationary at the flower head while drinking. Scent-marking was scored when a male walked on a flower or branch, often making repeated passes over the surface without drinking. Patrolling was scored when a male engaged in slow, looping or hovering inspection flights around flowers or branches without landing.

For each individual, the total number of events assigned to each behavior was extracted and used to calculate behavioral composition and within-individual rank order. Agreement between manual and automated classifications was assessed using these biologically meaningful summaries (Tables S1–S2).

First, behavioral composition was compared by calculating, for each individual, the proportion of events classified as patrolling, scent-marking, and feeding by manual scoring and by the algorithm (Table S1). Behavioral proportions were closely matched between methods for three of the four individuals. Larger discrepancies were observed for one individual (ID 41), for which the automated classification assigned a higher proportion of patrolling events and lower proportions of feeding and scent-marking events compared with manual scoring.

**Table S1. Agreement in behavioral composition between manual scoring and automated classification (per bee).**

| **id_no** | **behavior** | **n_manual** | **prop_manual** | **n_algo** | **prop_algo** | **treatment** | **abs_diff** | **signed_diff** |
| --- | --- | --- | --- | --- | --- | --- | --- | --- |
| 10 | patrolling | 55 | 0.809 | 49 | 0.831 | dispersed_high | 0.022 | 0.022 |
| 10 | scent_marking | 13 | 0.191 | 10 | 0.169 | dispersed_high | 0.022 | -0.022 |
| 10 | feeding | 0 | 0 | 0 | 0 | dispersed_high | 0 | 0 |
| 18 | patrolling | 40 | 0.889 | 36 | 0.837 | clumped_high | 0.052 | -0.052 |
| 18 | scent_marking | 1 | 0.022 | 1 | 0.023 | clumped_high | 0.001 | 0.001 |
| 18 | feeding | 4 | 0.089 | 6 | 0.140 | clumped_high | 0.051 | 0.051 |
| 39 | patrolling | 4 | 1 | 5 | 1 | clumped_low | 0 | 0 |
| 39 | scent_marking | 0 | 0 | 0 | 0 | clumped_low | 0 | 0 |
| 39 | feeding | 0 | 0 | 0 | 0 | clumped_low | 0 | 0 |
| 41 | patrolling | 13 | 0.333 | 25 | 0.676 | dispersed_low | 0.342 | 0.342 |
| 41 | scent_marking | 16 | 0.410 | 10 | 0.270 | dispersed_low | 0.140 | -0.140 |
| 41 | feeding | 10 | 0.256 | 2 | 0.054 | dispersed_low | 0.202 | -0.202 |

For each bee (*id_no*) and behavior (*patrolling*, *scent_marking*, *feeding*), *n_manual* is the number of manually scored events and *prop_manual* is the proportion of that bee’s manually scored events in that behavior. *n_algo* and *prop_algo* are the corresponding count and proportion from the automated classifier, computed over the same time window. *treatment* indicates the experimental condition for that bee. *abs_diff* is the absolute difference in proportions (*|prop_algo − prop_manual|*), and *signed_diff* is the signed difference (*prop_algo − prop_manual*).

Second, agreement in behavioral rank order within individuals was assessed using Spearman’s rank correlation and qualitative rank comparisons (Table S2). Manual and automated classifications produced identical rank orders of behavioral proportions for three individuals, resulting in perfect rank agreement. For the remaining individual (ID 41), rank agreement was partial, reflecting the differences in estimated behavioral composition described above.

**Table S2. Agreement in within-individual rank order of behaviors between manual scoring and automated classification.**

| **id_no** | **spearman_rho** | **manual_rank** | **algo_rank** | **treatment** |
| --- | --- | --- | --- | --- |
| 10 | 1 | patrolling > scent_marking > feeding | patrolling > scent_marking > feeding | dispersed_high |
| 18 | 1 | patrolling > feeding > scent_marking | patrolling > feeding > scent_marking | clumped_high |
| 39 | 1 | patrolling > scent_marking > feeding | patrolling > scent_marking > feeding | clumped_low |
| 41 | 0.5 | scent_marking > patrolling > feeding | patrolling > scent_marking > feeding | dispersed_low |

*spearman_rho* is Spearman’s rank correlation between the manual and automated behavioral proportions across the three behaviors (feeding, scent-marking, patrolling) for each bee. *manual_rank* and *algo_rank* show the qualitative rank ordering of behaviors from highest to lowest proportion. *treatment* indicates the experimental condition for that bee.

Together, these analyses indicate that the automated behavioral classification reliably reproduces individual-level behavioral composition and rank-order relationships among behaviors, while also revealing limited cases of individual-level divergence.

**Robustness of behavioral classification and statistical analyses**

To evaluate whether our conclusions were sensitive to the thresholds used to define feeding, scent-marking, and patrolling, we performed a ±20% perturbation of the two key classification parameters (minimum flight speed and distance-from-item). After each perturbation, all trajectories were reclassified and the full statistical workflow was re-run.

A qualitative summary of model outcomes across all perturbations is provided in Table S3.

**Speed threshold (±20%)**

Varying the minimum flight-speed threshold (original: 70 mm/s; perturbed: 56–84 mm/s) had minimal impact on behavioral detection (Fig. S1). Across all behaviors, median changes in event number or duration remained <3.3%.

Re-running the statistical analyses produced qualitatively identical outcomes:

- Behavior, Array, and Behavior × Array remained significant.
- Nectar availability was consistently non-significant.
- Weak interactions appearing at extreme perturbations never altered interpretation (Table S3).

**
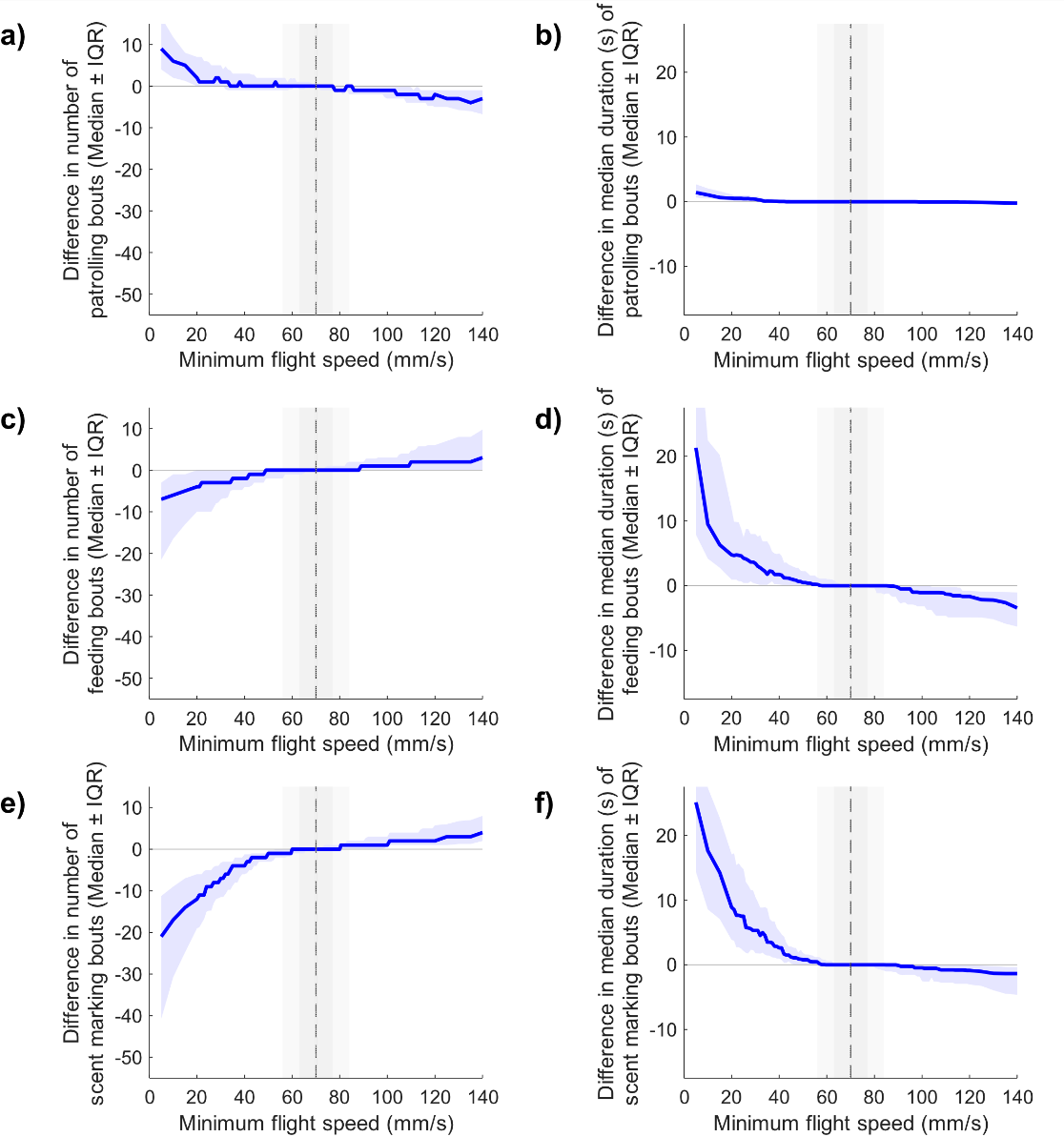
**

**Fig S1: Sensitivity of behavioral classifications to changes in minimum flight speed parameter.** (a) Blue line shows median change in number of patrolling bouts detected with variation of the cutoff speed that determines whether movement is categorized as flight or walking/sitting, as compared to the number of bouts detected using the value chosen in the main text (70 mm/s = 7 cm/s). Pale blue shaded region shows the interquartile range. Grey horizontal line indicates zero change in number of bouts. Vertical dashed line shows the value of the minimum flight speed parameter used in the main text (70 mm/s = 7 cm/s). Pale grey shaded region indicates flight speeds ±20% of value used in main text; darker grey shaded region indicates flight speeds ±10% of value used in main text. Other panels show the effects of varying the flight speed parameter on: (b) median duration of patrolling bouts, (c) number of visits to artificial flowers involving feeding, (d) median duration of feeding visits, (e) number of visits to artificial flowers or branches involving scent-marking behavior, (f) median duration of scent-marking visits.

**Distance threshold (±20%)**

Modifying the spatial distance threshold (original: 200 mm; perturbed: 160–240 mm) had similarly negligible effects (Fig. S2). Feeding and scent-marking classifications were extremely stable (<3.2% variation), reflecting their spatially constrained nature. Patrolling increased slowly with threshold size but varied by only ±3–5%.

Statistical outcomes remained unchanged across perturbations (Table S3):

- All main effects and key interactions preserved significance and direction.
- Weak Array × Nectar interactions appeared only at the lowest threshold but did not affect conclusions.


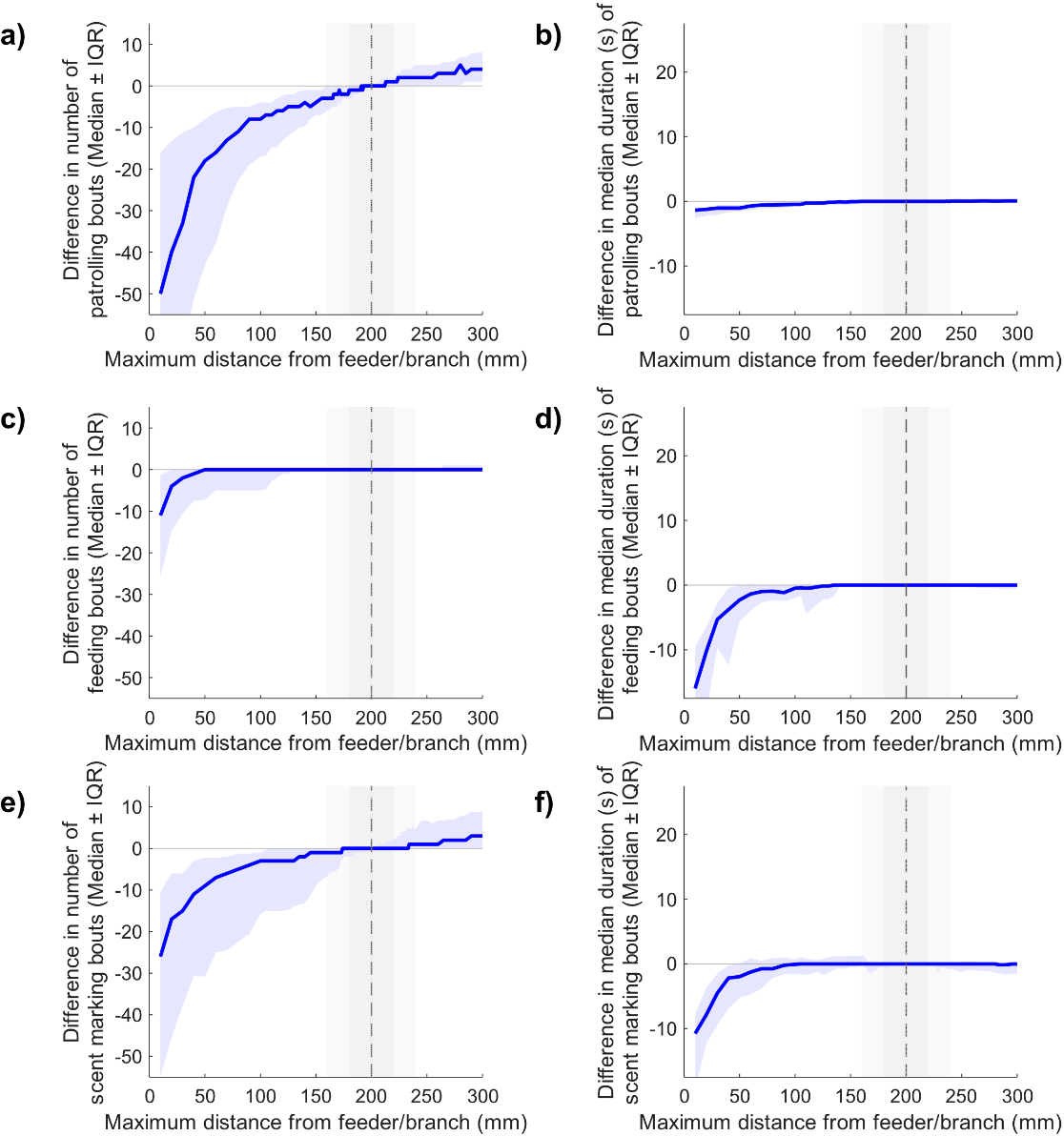


**Fig S2: Sensitivity of behavioral classifications to changes in maximum distance parameter.** (a) Blue line shows median change in number of patrolling bouts detected with variation of the distance from an artificial flower or branch within which a bee must approach for any behavior to be considered patrolling, feeding or scent-marking, as compared to the number of bouts detected using the value used in the main text (200 mm = 20 cm). Pale blue shaded region shows the interquartile range. Grey horizontal line indicates zero change in number of bouts. Vertical dashed line shows the value of the distance parameter chosen in the main text (200 mm = 20 cm). Pale grey shaded region indicates distance ±20% of value used in main text; darker grey shaded region indicates distance ±10% of value used in main text. Other panels show the effects of varying the distance parameter on: (b) median duration of patrolling bouts, (c) number of visits to artificial flowers involving feeding, (d) median duration of feeding visits, (e) number of visits to artificial flowers or branches involving scent-marking behavior, (f) median duration of scent-marking visits.

**Table S3. Robustness of statistical outcomes under ±20% variation in classification thresholds**

**1. Behavioral Frequency Model**

*Model: Behavior + Array + Nectar + Behavior × Array*

| **Effect** | **Original Thresholds** | **Speed ±20%** | **Distance ±20%** | **Interpretation** |
| --- | --- | --- | --- | --- |
| **Behavior** | ✓ | ✓ | ✓ | Stable across thresholds |
| **Array (Spatial distribution)** | ✓ | ✓ | ✓ | Stable across thresholds |
| **Nectar** | NS | NS | NS | Always non-significant |
| **Behavior × Array** | ✓ | ✓ | ✓ | Stable and unchanged |
| **Behavior × Nectar** | — | — | ≈ (weak, marginal at –20%) | Does not affect conclusions |
| **Array × Nectar** | — | — | ≈ (weak, marginal at –20%) | Does not affect conclusions |

**2. Total Behavioral Duration Model**

*Model: Behavior + Array + Nectar + Behavior × Array + Nectar × Array*

| **Effect** | **Original Thresholds** | **Speed ±20%** | **Distance ±20%** | **Interpretation** |
| --- | --- | --- | --- | --- |
| **Behavior** | ✓ | ✓ | ✓ | Dominant, unchanged |
| **Array (Spatial distribution)** | ✓ | ✓ | ✓ | Stable |
| **Nectar** | NS | NS | NS | Always non-significant |
| **Behavior × Array** | ✓ | ✓ | ✓ | Stable |
| **Array × Nectar** | ≈ (weak) | ≈ (weak) | ≈ (weak) | Small interaction; interpretation unchanged |

**3. Per-Event Duration Model**

*Model: Behavior + Array + Nectar + Behavior × Array + Nectar × Array*

| **Effect** | **Original Thresholds** | **Speed ±20%** | **Distance ±20%** | **Interpretation** |
| --- | --- | --- | --- | --- |
| **Behavior** | ✓ (very strong) | ✓ | ✓ | Dominant and unchanged (Feeding > Scent-marking > Patrolling) |
| **Array (Spatial distribution)** | NS | NS | NS / very weak | No meaningful effect |
| **Nectar** | NS | NS | NS | No effect |
| **Behavior × Array** | ✓ | ✓ | ✓ | Stable and unchanged |
| **Array × Nectar** | ≈ (weak) | ≈ (weak) | ≈ (weak) | Minor; does not affect interpretation |
| **Behavior × Nectar** | — | — | NS | — |
| **Array × Nectar × Behavior** | — | ≈ (appears, small) | ≈ (appears, small) | Trivial; conclusions unchanged |

**Summary:**
Across both speed and distance perturbations, no qualitative result changed. All main conclusions in the manuscript are fully robust to uncertainty in classification thresholds.

**Speed and angular velocity thresholds of patrolling bouts (±20%)**

To assess sensitivity of patrolling classification to the chosen movement thresholds, we varied the maximum flight speed (50 cm/s) and minimum angular velocity (125°/s) used to define patrolling by ±10% and ±20% (Fig. S3). Varying the speed threshold resulted in modest changes in the number of detected patrolling bouts (median change ≤ ±7.4%), with the chosen value lying close to an inflection point beyond which increasing the threshold added few additional bouts. In contrast, patrolling bout duration was effectively insensitive to changes in either parameter. Varying the angular velocity threshold had minimal effects on both the number and duration of patrolling bouts (≤ ±2%). These analyses confirm that patrolling classification is robust to reasonable variation in threshold values and not driven by arbitrary parameter choices.

**
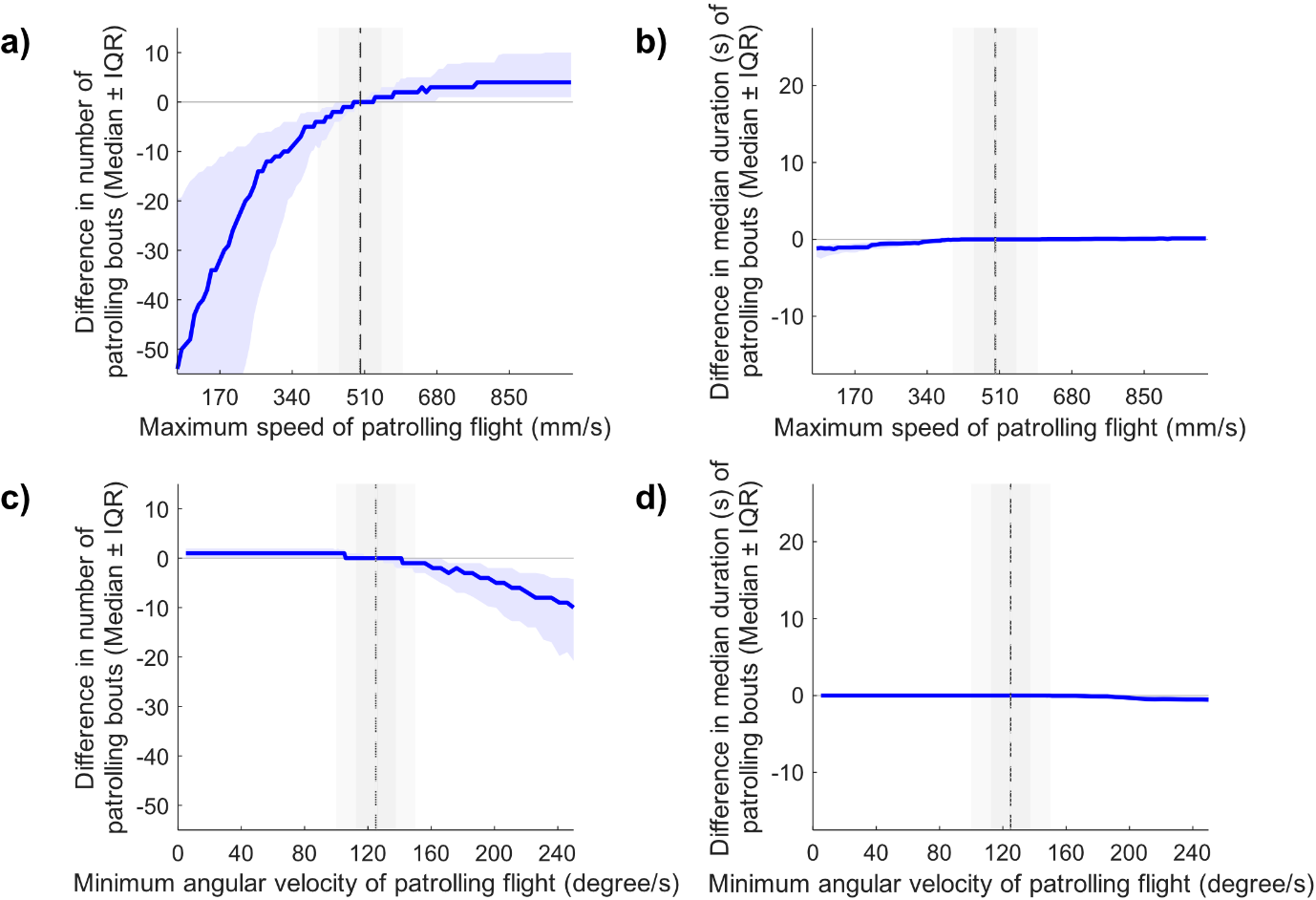
**

**Fig S3: Sensitivity of patrol behavior classifications to changes in parameters.** (a) Blue line shows median change in number of patrolling bouts detected with variation of the maximum flight speed for movement to be classified as patrolling, as compared to the number of bouts detected using the value chosen in the main text (500 mm/s = 50 cm/s). Pale blue shaded region shows the interquartile range. Grey horizontal line indicates zero change in number of bouts. Vertical dashed line shows the value of the minimum flight speed parameter used in the main text (500 mm/s = 50 cm/s). Pale grey shaded region indicates flight speeds ±20% of value used in main text; darker grey shaded region indicates flight speeds ±10% of value used in main text. (b) Effect of varying the same maximum speed parameter on median duration of patrolling bouts. (c) Median change in number of patrolling bouts detected with variation in the minimum angular velocity necessary for movement to be classified as patrolling (value used in main text: 125°/s). (d) Effect of varying the same minimum angular velocity on median duration of patrolling bouts.

**Model selection tables**

**Table S4. Model selection for behavioral frequency (log-transformed).** Candidate linear mixed-effects models were compared using AIC, with Male ID included as a random intercept in all models. The response variable was log-transformed behavioral frequency. Model selection followed a stepwise simplification from the global model. The intercept-only model is included for reference.

| **Model** | **Fixed effects** | **k** | **AIC** | **Delta AIC** | **AIC weight** |
| --- | --- | --- | --- | --- | --- |
| M1 | Behavior + Array + Nectar + Behavior × Array | 9 | 439.91 | 0.00 | 0.440 |
| M2 | Behavior + Array + Nectar + Behavior × Array + Array × Nectar | 10 | 440.24 | 0.33 | 0.372 |
| M3 | Behavior + Array + Nectar + Behavior × Array + Behavior × Nectar + Array × Nectar | 12 | 442.45 | 2.54 | 0.123 |
| M4 | Behavior × Array × Nectar (global model) | 14 | 443.72 | 3.82 | 0.065 |
| M5 | Intercept-only model | 3 | 474.77 | 34.87 | 0.000 |

**Table S5. Model selection for total behavioral duration (log-transformed).** Candidate linear mixed-effects models were compared using AIC, with Male ID included as a random intercept in all models. The response variable was log-transformed duration. The global model included all main effects and interactions. The intercept-only model is included for reference.

| **Model** | **Fixed effects** | **k** | **AIC** | **Delta AIC** | **AIC weight** |
| --- | --- | --- | --- | --- | --- |
| M1 | Behavior + Nectar + Array + Behavior × Array + Nectar × Array | 10 | 401.65 | 0.00 | 0.754 |
| M2 | Behavior + Nectar + Array + Behavior × Nectar + Behavior × Array + Nectar × Array | 12 | 404.38 | 2.74 | 0.192 |
| M3 | Behavior × Nectar × Array (global model) | 14 | 406.90 | 5.26 | 0.054 |
| M4 | Intercept-only model | 3 | 425.98 | 24.34 | 0.000 |

**Table S6. Model selection for behavioral duration per event (log-transformed).** Candidate linear mixed-effects models were compared using AIC, with Male ID included as a random intercept in all models. The response variable was log-transformed event duration. Model selection followed a stepwise simplification from the global model. The intercept-only model is included for reference.

| **Model** | **Fixed effects** | **k** | **AIC** | **Delta AIC** | **AIC weight** |
| --- | --- | --- | --- | --- | --- |
| M1 | Behavior + Nectar + Array + Behavior × Array + Nectar × Array | 10 | 29626.22 | 0.00 | 0.812 |
| M2 | Behavior + Nectar + Array + Behavior × Nectar + Behavior × Array + Nectar × Array | 12 | 29629.80 | 3.58 | 0.136 |
| M3 | Behavior × Nectar × Array (global model) | 14 | 29631.69 | 5.47 | 0.053 |
| M4 | Intercept-only model | 3 | 37002.85 | 7376.62 | 0.000 |

**Table S7. Model selection for return cycle mean.** Candidate linear mixed-effects models were compared using AIC, with Male ID included as a random intercept in all models. The response variable was log-transformed event duration. Model selection followed a stepwise simplification from the global model. The intercept-only model is included for reference.

| **Model** | **Fixed effects** | **k** | **AIC** | **Delta AIC** | **AIC weight** |
| --- | --- | --- | --- | --- | --- |
| M1 | Time x Nectar | 6 | 830.10 | 0.00 | 0.834 |
| M2 | Intercept-only model | 3 | 834.80 | 4.69 | 0.080 |
| M3 | Time | 4 | 836.28 | 6.19 | 0.038 |
| M4 | Time x Nectar x Array (global model) | 10 | 836.29 | 6.19 | 0.038 |
| M5 | Time x Array | 6 | 838.89 | 8.80 | 0.010 |

**Table S8. Model selection for immediate revisits.** Candidate negative binomial mixed-effects models were compared using AIC, with Male ID included as a random intercept in all models. Model selection followed a stepwise simplification from the global model. The intercept-only model is included for reference.

| **Model** | **Fixed effects** | **k** | **AIC** | **Delta AIC** | **AIC weight** |
| --- | --- | --- | --- | --- | --- |
| M1 | Visit block x Behavior x Array | 14 | 2535.56 | 0.00 | 0.890 |
| M2 | Visit block x Behavior x Array x Nectar (global model) | 26 | 2539.75 | 4.19 | 0.110 |
| M3 | Visit block x Behavior | 8 | 2600.69 | 65.13 | 6.407e-15 |
| M4 | Visit block | 4 | 2666.76 | 131.20 | 2.887e-29 |
| M5 | Intercept-only model | 3 | 2680.49 | 144.93 | 3.007e-32 |
